# Efficacy and safety of hepatic arterial infusion chemotherapy combined with programmed cell death protein-1 antibody and lenvatinib for advanced hepatocellular carcinoma

**DOI:** 10.3389/fmed.2022.919069

**Published:** 2022-09-01

**Authors:** Yongkang Xu, Shumin Fu, Ye Mao, Shenglan Huang, Dan Li, Jianbing Wu

**Affiliations:** Department of Oncology, The Second Affiliated Hospital of Nanchang University, Nanchang, China

**Keywords:** hepatic arterial infusion chemotherapy, PD-1 antibody, lenvatinib, hepatic arterial infusion chemotherapy, hepatocellular carcinoma

## Abstract

**Background:**

The purpose of the study was to assess the efficacy and safety in patients with advanced hepatocellular carcinoma (HCC) who are undergoing hepatic arterial infusion chemotherapy (HAIC) combined with programmed cell death protein-1 (PD-1) antibody and lenvatinib.

**Methods:**

We retrospectively evaluated 61 patients treated with HAIC combined with PD-1 antibody and lenvatinib at the Second Affiliated Hospital of Nanchang University between September 2020 and January 2022 for advanced HCC. We analyzed tumor response, progression free survival (PFS), and treatment-related adverse events (TRAEs).

**Results:**

The objective response rate (ORR) was 36.1% (RECIST 1.1)/57.4% (mRECIST) and the disease control rate (DCR) was 82.0%. The overall median PFS was 6.0 months, 6.7 months for first-line treatment, and 4.3 months for second-line treatment. The most common TRAEs were neutropenia (50.8%), abdominal pain (45.9%), and aspartate aminotransferase increase (39.3%).

**Conclusion:**

Hepatic arterial infusion chemotherapy combined with PD-1 antibody and lenvatinib is effective in the treatment of advanced HCC, and the TRAEs are generally controllable.

## Introduction

Primary liver cancer is the third leading cause of cancer-related deaths worldwide in 2020, with about 830,000 deaths annually, and hepatocellular carcinoma (HCC) accounts for 85–90% of primary liver cancers (1). Despite the surveillance programs in high-risk patients, the majority of patients with HCC are diagnosed at an advanced stage and have lost the chance for curative surgery, with a median survival time of only 4.2 to 7.9 months (2). Although there are many treatment methods for advanced unresectable HCC (such as interventional therapy, radiotherapy, immunotherapy, targeted therapy, and so on) and a single treatment method has achieved certain curative effect, the improvement in overall survival is still not satisfactory.

In the recent years, significant progress has been made in the systematic treatment of advanced HCC. However, the efficiency of molecular targeted agent (MTA) or immune checkpoint inhibitors (ICIs) alone is relatively limited, and its ORR is less than 20% (3). Recent studies showed that MTA combined with ICIs can enhance the immunogenicity of tumor cell and reshape the tumor microenvironment, showing a synergistic effect of “1 + 1 > 2” (4). Moreover, it is worth mentioning that IMbrave150 III study (atezolizumab combined with bevacizumab) has increased ORR to 27.3% (5). Therefore, the ORR of combination MAT with ICIs will continue to rise in the future. Hepatic arterial infusion chemotherapy (HAIC) is a common treatment modality for advanced hepatocellular carcinoma (HCC), particularly in Asia. According to Japan Society of Hepatology (JSH) guideline, HAIC was recommended as a standard therapy for HCC with portal vein tumor thrombus (6). Recently, a randomized clinical trial by MinHe et al. showed better outcomes with HAIC (FOLFOX) plus sorafenib than with sorafenib alone for HCC with portal vein invasion, and the combination therapy resulted in nearly two times longer than did sorafenib monotherapy (13.37 vs. 7.13 months, *p* < 0.001) (7). At the 2020 European Society for Medical Oncology (ESMO) meeting, HAIC compared with transarterial chemoembolization (TACE) in the treatment of unresectable HCC with maximum diameter ≥ 7 cm, the PFS and OS in HAIC group, was longer than TACE group (9.63 vs. 5.40 months, *p* < 0.001; 23.1 vs. 16.07 months, *p* < 0.001), and there were more hepatic resection as conversion therapy in HAIC group (23.8 vs. 11.55%, *p* = 0.004) (8). Furthermore, more and more studies have revealed that HAIC-based local therapy combined with immunotherapy and targeted therapy has an excellent therapeutic effect in patients with advanced HCC. Recently, the combined therapy of apatinib, toripalimab, and HAIC has been reported as an abstract in the American Society of Clinical Oncology meeting, with a response rate of 100%; however, only six patients were included in the analysis (9). He et al. evaluated the efficacy and safety of HAIC plus lenvatinib and toripalimab as first-line treatment in advanced HCC (10). The outcome of 71 patients with HCC who underwent HAIC combined with lenvatinib and toripalimab (LeToHAIC group) compared with 86 patients who received lenvatinib alone showed that the median PFS in the LeToHAIC group was significantly longer than that in the lenvatinib group (11.1 vs. 5.1 months *p* < 0.001), and the DCR and ORR were also significantly higher in the LeToHAIC group than those in the lenvatinib group based on the mRECIST criteria (90.1 vs. 72.1%, *p* = 0.005; 67.6 vs. 16.3%, *p* < 0.001). In terms of safety, the majority of TRAEs are grades 1–2, and the grades 3–4 TRAEs group are higher, but only 8.5%. Hypertension and increased aspartate aminotransferase are the most common TRAEs, both of which are generally safe and controllable. According to the abovementioned data, the combination treatment has demonstrated promising antitumor efficacy and tolerable safety in patients with advanced HCC and has a greater surgical conversion rate.

In conclusion, the triple combination therapy of HAIC, programmed cell death protein-1 (PD-1) inhibitor, and MTA has yielded a promising clinical efficacy and safety in patients with advanced HCCC. This study will further explore the real-world treatment effect and provide more evidence for clinical practice.

## Materials and methods

### Patients and data collection

We retrospectively reviewed 106 patients treated with HAIC combined system therapy at the Second Affiliated Hospital of Nanchang University between September 2020 and January 2022 for advanced HCC. Finally, 61 patients who received HAIC combined PD-1 antibody and lenvatinib were included in this study. The baseline data of patients, including patient’s age, gander, tumor stage, HBV infection, ECOG PS, laboratory data, pervious therapy, hepatic reserve (Child–Pugh score), imaging data (vascular invasion and extrahepatic lesion), and adverse reaction, were collected, and we gathered information from patients’ medical records and followed up with them over the phone.

The eligibility criteria were as follows: (1) The diagnosis of HCC was based on the pathological findings or according to the guidelines of the American Association for the Study of Liver Disease (AASLD); (2) patients had a tumor classification of Barcelona Clinic Liver Cancer (BCLC) B or C and are considered unsuitable for curative surgery therapy; (3) the Eastern Cooperative Oncology Group performance status (ECOG PS) of 2 or less; (4) Child–Pugh (CP) liver function class A or B; and (5) patients had at least one cycles of HAIC combined PD-1 and lenvatinib. The exclusion criteria included the following: (1) the presence of serious cardiopulmonary or hepatorenal failure; (2) with another previous or current malignant tumors; and (3) the survival time is estimated to be less than 3 months.

### Treatment

Hepatic artery infusion chemotherapy usually adopts the Seldinger technique to puncture the femoral artery. The catheter is placed in the celiac trunk or common hepatic artery for digital subtraction angiography (DSA). If necessary, angiography of superior mesenteric artery, phrenic artery, left gastric artery, and right renal artery shall be performed to find the blood supply of the tumor. Then, a microcatheter was inserted into the proper hepatic artery for chemoinfusion. The therapeutic regimen is modified FOLFOX6 regimens including oxaliplatin (85 mg/m^2^ infusion for 3 h on day 1), leucovorin 400 mg/m^2^ from 3 to 5 h on day 1), and 5-fluorouracil (bolus 400 mg/m^2^ and then 2,400 mg/m^2^ for 46 h). This treatment was repeated every 3 weeks until unacceptable toxicity or the patients refused the treatment.

PD-1 inhibitor and lenvatinib were treated within 7 days after the HAIC. The PD-1 inhibitor includes pembrolizumab, camrelizumab, tislelizumab, sintilimab, and toripalimab. PD-1 antibody was administered for each HAIC treatment and every 3 weeks after HAIC was discontinued until intrahepatic lesions progression or unacceptable toxicity. Patients received oral lenvatinib 8 mg/day (for bodyweight < 60 kg) or 12 mg/day (for bodyweight > 60 kg). If the patient cannot tolerate the lenvatinib-related toxicities, the dose can be reduced to 8 or 4 mg/day.

### Follow-up and assessments

During the treatment period, all patients were followed up with routine examinations, which were collected within 1 week before the initial treatment and subsequently conducted every 3 ± 1 week. Moreover, each patient must have at least one measurable target lesion, and the efficacy of combination therapy was assessed every 8–12 weeks by dynamic CT or dynamic MRI during the treatment period. The tumor response and progression were determined by both RECIST version 1.1 and mRECIST. Tumor response was defined as complete remission (CR), partial remission (PR), disease stability (SD), or disease progression (PD). Overall response rate (ORR) was calculated as the sum of CR and PR. Disease control rate (DCR) was calculated as the sum of CR, PR, and SD. Progression-free survival (PFS) was calculated from the first day of HAIC until the date of disease progression, death, or last day follow-up and overall survival (OS) was calculated from the first date of HAIC to the date of death or the last day of the follow-up period. The toxicities were assessed based on the Common Terminology Criteria for Adverse Everts (CTCAE) version 5.0.

### Statistical analysis

The count data are expressed in absolute numbers and/or percentages. The measurement data conforming to the normal distribution are described by mean ± standard error, the measurement data not conforming to the normal distribution are described by median (range), and the comparison of not conforming to the normal distribution within the group is carried out by a non-parametric test. Survival curves were calculated by Kaplan–Meier method, and log-rank test was used to analyze the differences between groups. Cox regression models were used for univariate analysis and multivariate analysis to determine the prognostic factors for the PFS. *p*-value < 0.05 was considered to suggest significant difference. The data were assessed using SPSS v25.0 software, R software v3.6.3, and GraphPad Prism v8.0 for analysis.

## Results

### Patients

**FIGURE 1:**
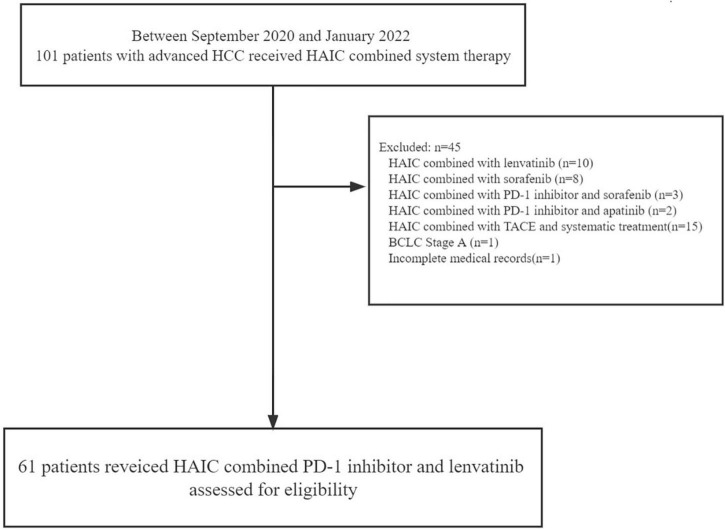
Patient selection flow. HCC, hepatocellular carcinoma; HAIC, hepatic arterial infusion chemotherapy; PD-1, programmed cell death protein-1; TACE, transarterial chemoembolization; BCLC, Barcelona Clinic Liver Cancer.

From September 2020 to January 2022, we screened 106 patients with advanced HCC at the Second Affiliated Hospital of Nanchang University between September 2020 and January 2022 for advanced HCC receiving HAIC combined system therapy. We ruled out 45 patients in our cohort, as there were 10 patients using HAIC combined with lenvatinib, 8 patients using HAIC combined with sorafenib, 3 patients using HAIC combined with PD-1 inhibitor and sorafenib, 2 patients using HAIC combined with PD-1 inhibitor and apatinib, 15 patients using HAIC combined with TACE and systematic treatment, 1 patient using BCLC stage A, and patients using of missing data. Hence, we recruited 61 patients using HAIC, PD-1 antibody, and lenvatinib altogether in our study (Figure 1).

The characteristics of clinical baseline data of 61 patients are summarized in Table 1. Mean ages of patients were 52.6 (SD 12.6) years and more than 85% of patients were men. At the start of the study, the majority of the participants were classified as Child–Pugh A (86.9%). Furthermore, 52 (85.2%) had BCLC stage C and 46 (75.4%) had portal vein tumor thrombus, 58 (95.1%) had hepatitis B virus (HBV) infection and most patients had varied degrees of liver cirrhosis, and 22 (36.1%) had tumor diameters higher than 10 cm. In this study, the cycles of PD-1 antibody plus lenvatinib ranged from 1 to 22, with a median of 4, and the cycles of HAIC ranged from 1 to 5, with a median of 2. The PD-1 antibody categories in each group are summarized in Table 2, including pembrolizumab (1), camrelizumab (37), tislelizumab (12), sintilimab (9), and toripalimab (2). After termination of the combination therapy, 23 patients received subsequent treatments, which are summarized in Table 3.

**TABLE 1:** Baseline clinical characteristics of patients.

Characteristics *n* (%)	Patient number (%)
**Age**	
≥60	22 (36.1%)
<60	39 (63.9%)
**Gender**	
Male	53 (86.9%)
Female	8 (13.1%)
**ECOG PS**	
0	13 (21.3%)
1	41 (67.2%)
2	7 (11.5%)
**Child-Pugh**	
A	53 (86.9%)
B	8 (13.1%)
**BCLC**	
B	9 (14.8%)
C	52 (85.2%)
**Etiology of HCC**	
HBV	58 (95.1%)
Non-HBV	3 (4.9%)
**Liver cirrhosis**	
YES	48 (78.7%)
NO	13 (21.3%)
**Portal vein tumor thrombus**	
YES	46 (75.4%)
NO	15 (24.6%)
**Extrahepatic spread**	
YES	44 (72.1%)
NO	17 (27.9%)
**Tumor size, cm**	
≥10	22 (36.1%)
<10	39 (63.9%)
**Baseline AFP, ng/mL**	
≥400	38 (62.3%)
<400	23 (37.7%)
**Treatment lines**	
First line	47 (77.0%)
Second line	14 (23.0%)

BCLC, barcelona clinic liver cancer; ECOG PS, eastern cooperative oncology group performance status; HBV, hepatitis B virus; AFP, alpha fetoprotein; HCC, hepatocellular carcinoma; TACE, transarterial chemoembolization.

**TABLE 2:** The type of immune checkpoint inhibitors (ICIs).

Drug	Patient numbers (%)
Camrelizumab	37 (60.7%)
Pembrolizumab	1 (1.6%)
Sintilimab	9 (14.8%)
Toripalimab	2 (3.3%)
Tislelizumab	12 (19.7%)

**TABLE 3:** Number of patients who received subsequent treatments.

Types of subsequent treatments	Patient numbers
Surgical resection	2
Radiotherapy	3
Radiofrequency ablation	2
TAEC combined PD-1 inhibitor and sorafenib	2
TACE combined PD-1 inhibitor and regorafenib	2
Atezolizumab plus bevacizumab	1
PD-1 inhibitor plus sorafenib	1
PD-1 plus regorafenib	1
PD-1 inhibitor plus apatinib	1
Donafenib monotherapy	1
Optimal supportive treatment	3

TACE, transarterial chemoembolization; PD-1, programmed cell death protein-1.

### Efficacy

**FIGURE 2:**
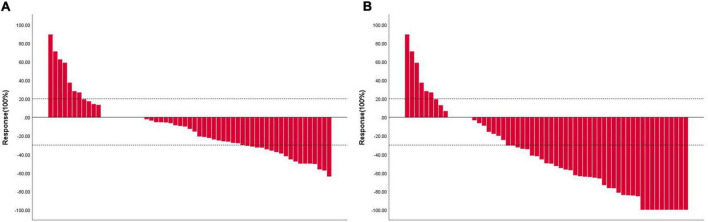
Waterfall plot showing maximum changes in tumor size of patients receiving HAIC combined lenvatinib and PD-1 antibody. **(A)** Assessed with RECIST 1.1 in patients with image measurements before and after treatment; **(B)** assessed with mRECIST in patients with image measurements before and after treatment. RECIST, Response Evaluation Criteria in Solid Tumors; mRECIST, modified Response Evaluation Criteria in Solid Tumors; HAIC, hepatic arterial infusion chemotherapy; PD-1, programmed cell death protein-1.

**FIGURE 3:**
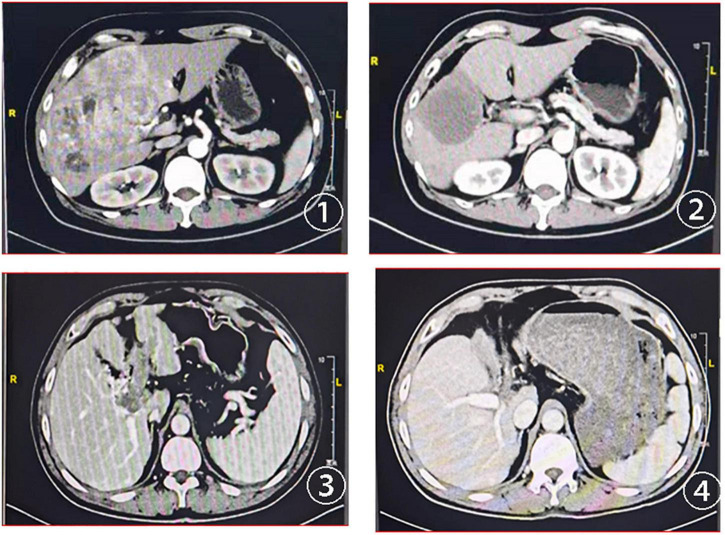
CT scans at baseline and after treatment assessment for two advanced patients with HCC treated with HAIC combined lenvatinib and PD-1. ① Baseline CT scans of the first patient; ② CT scans of the first patient after two cycles of the combined treatment; ③ baseline CT scans of the second patient with portal vein tumor thrombus; ④ the portal vein tumor thrombus disappeared after the combined treatment.

In this study, the deadline for follow-up was 1 March 2022 and the median follow-up time was 6.0 months. The tumor response evaluation of 61 patients is shown in Table 4. A waterfall plot was constructed to show maximum changes in tumor size of patients receiving HAIC combined lenvatinib and PD-1 antibody (Figure 2) and favorable tumor response of patient with large hepatocellular carcinoma or main portal vein tumor thrombus hepatocellular carcinoma treated by HAIC combined lenvatinib and PD-1 antibody (Figure 3). Based on the RECIST criteria, there are 22 patients with partial response (PR), 28 stable disease (SD), and 9 progressive diseases (PD), which indicates that the ORR and DCR were 36.1 and 82.0%, respectively. In the first-line treatment, there were 21 (44.7%) patients with PR, 18 (38.3%) with SD, and 6 (12.8%) with PD, and the ORR and DCR were 44.7 and 83.0%. In the second-line group, there were 1 (7.1%) patient with PR, 10 (71.4%) with SD, and 3 (21.4%) with PD, indicating that the ORR and DCR were 7.1 and 78.6%. Based on the mRECIST criteria, there were 10 (16.4%) patients with complete response, 25 (41.0%) with PR, 15 (24.6%) with SD, and 9 (14.8%) with PD, indicating that the ORR and DCR were 57.4 and 82.0%. In the first-line treatment, there were 9 (19.1%) patients with CR, 22 (46.8) with PR, 8 (17.0%) with SD, and 6 (12.8%) with PD, and the ORR and DCR were 66.0 and 83.0%. In the second-line group, there were 1 (7.1%) patient with CR, 3 (21.4%) with PR, 7 (50.0%) with SD, and 3 (21.4%) with PD, indicating that the ORR and DCR were 28.6 and 78.6%.

**TABLE 4:** Summary of best response.

	RECIST1.1	mRECIST
**Overall response**		
Complete response	0 (0%)	10 (16.4%)
Partial response	22 (36.1%)	25 (41.0%)
Stable response	28 (45.9%)	15 (24.6%)
Progressive response	9 (14.8%)	9 (14.8%)
Not assessable	2 (3.3%)	2 (3.3%)
Overall response rate	22 (36.1%)	35 (57.4%)
Disease control rate	50 (82.0%)	50 (82.0%)
**Intrahepatic response**		
Complete response	0 (0%)	10 (16.4%)
Partial response	23 (37.7%)	27 (44.3%)
Stable response	28 (45.9%)	15 (24.6%)
Progressive response	8 (13.1%)	7 (11.5%)
Not assessable	2 (3.3%)	2 (3.3%)
Overall response rate	23 (37.7%)	37 (60.7%)
Disease control rate	51 (83.6%)	52 (85.2%)

RECIST, response evaluation criteria in solid tumor; mRECIST, modified response evaluation criteria in solid tumor.

**FIGURE 4:**
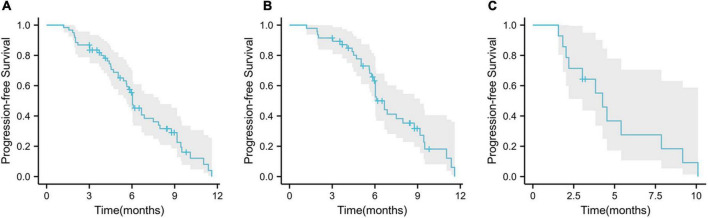
Kaplan–Meier curves for progression-free survival. **(A)** Overall population progression-free survival; **(B)** progression-free survival in first-line patients; **(C)** progression-free survival in second-line patients.

During the follow-up period, 45 (73.8%) patients had disease progression and 29 (47.5%) died. Among these patients, 33 (70.2%) had disease progression, 20 (42.6%) died in the first-line treatment, 12 patients (85.7%) developed disease, and 9 (64.3%) died in the second-line treatment. The median PFS of the whole population was 6.0 months (95% CI, 5.37–6.70; Figure 4A), the median PFS of first-line treatment was 6.7 months (95% CI, 6.07–7.27; Figure 4B), and the median PFS of second-line treatment was 4.3 months (95% CI, 3.20–5.33; Figure 4C).

**FIGURE 5:**
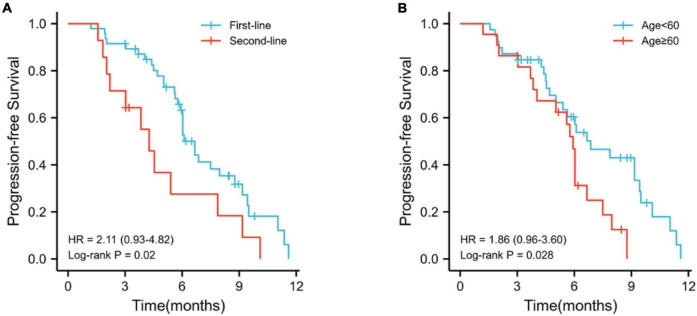
Kaplan–Meier survival analyses. **(A)** Kaplan–Meier curve for comparison of PFS between first-line treatment and second-line treatment; **(B)** Kaplan–Meier curve for comparison of PFS between old age (≥60) and young age (<60).

Kaplan–Meier survival analysis showed that the median PFS was longer in first-line treatment than second-line treatment (6.7 vs. 4.3 months, *p* = 0.2; Figure 5A) and patients younger than 6 years old had longer PFS than patients older than 60 years old (6.9 vs. 5.9 months, *p* = 0.2; Figure 5B). There were no significant differences in patient gender, tumor stage, HBV infection, ECOG PS score, portal vein tumor thrombus, extrahepatic metastasis, tumor size, the level of AFP (alpha-fetoprotein), Child–Pugh score, and liver cirrhosis (*p* > 0.05).

### Changes of liver function and APF level

**FIGURE 6:**
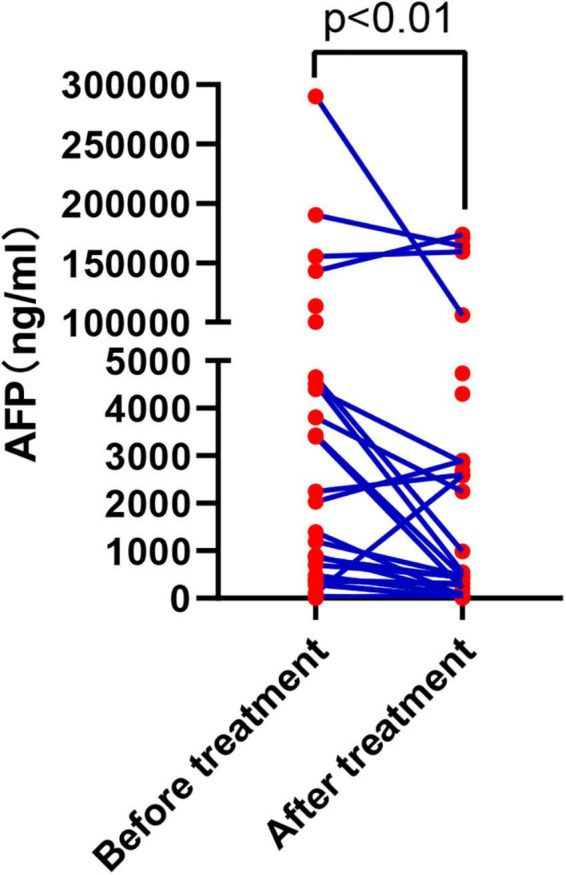
Variation in AFP level before and after the first combined treatment.

We assessed liver functional reserve within 7 days after the first HAIC treatment according to the Child–Pugh scoring system. Most patients maintained their previous hepatic reserve without further deterioration. After the first HAIC, liver function increased from CP-A to CP-B in 11 patients, and only one patient with CP-B experienced further deterioration. The APF levels were recorded after the first follow-up and compared to the baseline AFP. The level of APF in most individuals dropped following therapy, according to our study. The median AFP before treatment was 2,747.7 ng/ml, whereas the median AFP after treatment was 424.0 ng/ml, showing a statistically significant difference. We depict the changes of APF level in Figure 6.

### Safety

In this study, there were no treatment-related deaths, and the treatment-related adverse events (TRAEs) are presented in Table 5. Most patients (75.4%) experienced TRAEs following triple combination therapy with HAIC, PD-1 plus lenvatinib. The common TRAEs included neutropenia 50.8% (31/61), abdominal pain 45.9% (28/61), ALT increase 39.3% (24/61), thrombocytopenia 36.1% (22/61), hypertension 36.1% (22/61), AST increase 32.8% (20/61), hand foot syndrome 26.2% (16/61), rash 23.0% (14/61), and hypothyroidism 18.0% (11/61). Among the 61 patients, 16 (26.2%) experienced serious adverse events (grades 3–4). The common serious adverse reactions included abdominal pain 8.2% (5/61), neutropenia 6.6% (4/61), thrombocytopenia 4.9% (3/61), ALT increase 3.3% (2/61), and AST increase 3.3% (2/61).

**TABLE 5:** Summary of adverse events.

*n* (%)	All Grade	Grade 1	Grade 2	Grade 3–4
Fever	12 (19.7)	7 (11.5)	5 (8.2)	0
Abdominal pain	28 (45.9)	10 (18.0)	13 (21.3)	5 (8.2)
Nausea	20 (32.8)	11 (18.0)	9 (14.6)	0
Vomiting	8 (13.1)	5 (8.2)	3 (4.9)	0
Neutropenia	31 (50.8)	15 (24.6)	12 (19.7)	4 (6.6)
Thrombocytopenia	22 (36.1)	13 (21.3)	6 (9.8)	3 (4.9)
Elevated ALT Elevated AST	24 (39.3) 20 (32.8)	12 (19.7) 10 (16.4)	10 (16.4) 8 (13.1)	2 (3.3) 2 (3.3)
Hyperbilirubinemia	10 (16.4)	6 (9.8)	3 (4.9)	1 (1.6)
Hepatapostema	1 (1.6)	0	0	1 (1.6)
Rash	14 (23.0)	7 (11.5)	6 (9.8)	1 (1.6)
Hypertension	22 (36.1)	15 (24.6)	7 (11.5)	0
Diarrhea	7 (11.5)	4 (6.6)	3 (4.9)	0
Fatigue	9 (14.8)	3 (4.9)	6 (9.8)	0
Hoarseness	5 (8.2)	3 (4.9)	2 (3.3)	0
Bleeding	7 (11.5)	6 (9.8)	1 (1.6)	0
Hand–foot skin reaction	16 (26.2)	7 (11.5)	8 (13.1)	1 (1.6)
Cutaneous vascular hyperplasias	9 (14.8)	5 (8.2)	3 (4.9)	1 (1.6)
Hyperthyroidism	1 (1.6)	1 (1.6)	0	0
Hypothyroidism	11 (18.0)	10 (16.4)	1 (1.6)	0
ICIs-induced hepatitis	2 (3.3)	0	1 (1.6)	1 (1.6)
ICIs-induced pneumonia	1 (1.6)	0	1 (1.6)	0

ALT, alanine aminotransferase; AST, aspartate aminotransferase; ICIs, immune checkpoint inhibitors.

At the beginning of HAIC treatment, most patients had varying degrees of abdominal pain during the infusion of oxaliplatin. Usually stop the infusion or prolong the infusion time, the pain can be effectively relieved, whereas if patients have severe and acute abdominal pain, lidocaine can be used to relieve the pain. Then, we increased the oxaliplatin infusion time from 2 to 3 h, and the incidence of abdominal pain reduced significantly. In addition, one patient developed liver abscess following HAIC, which improved after puncture and drainage of the abscess.

The most common immune-related TRAE was grade 1–2 hypothyroidism (18.0%). A number of two patients experienced immune-related hepatitis, one developed immune-related pneumonia, one developed grade 3 immune-related rash, and one developed grade 3 skin capillary hyperplasia. After glucocorticoid therapy and a 2-week interruption of PD-1 antibody therapy, the liver function of two patients with immune-related hepatitis was recovered. After a month, the patient with immune-associated pneumonia recovered. After 2 months of glucocorticoid therapy and discontinued using immunotherapy, patients with immune-related dermatitis recovered. Patients with grade 3 cutaneous capillary hyperplasia improved after symptomatic support treatment and discontinuation of one cycle of immunotherapy.

The most common TRAE with lenvatinib was hypertension (36.1%), and one patient developed grade 3 hand foot syndrome, which improved 2 weeks after interval reduction.

### Prognostic factor analysis

The clinical characteristics were analyzed by univariate and multivariate Cox regressions to determine the prognostic factors related to PFS. The prognostic factors for PFS are listed in Table 6. Multivariate analysis identified that the number of treatment lines (first-line treatment vs. second-line treatment, HR = 3.215; 95% CI 1.520–6.951; *p* < 0.05) and age (<60 vs. ≥60, HR = 2.903; 95% CI 1.404–6.000; *p* < 0.05) were the independent risk factors for PFS.

**TABLE 6:** Univariate and multivariate analysis of risk factors for overall survival.

	Univariate analysis			Multivariate analysis		
	HR	95% CI	*P*-value	HR	95% CI	*P*-value
Sex (male/female)	1.419	0.594–3.392	0.431			
Age (<60 vs ≥60)	2.034	1.058–3.910	0.033	2.903	1.404–6.000	0.004
ECOG PS (0,1 vs 2)	0.601	0.232–1.556	0.294			
BCLC Stage (B vs C)	0.809	0.373–1.756	0.592			
Child-Pugh (A vs B)	1.732	0.723–4.174	0.218			
HBV (no vs yes)	0.437	0.132–1.447	0.176			
Liver cirrhosis (no vs yes)	1.167	0.515–2.645	0.712			
PVTT (no vs yes)	1.032	0.529–2.013	0.926			
Extrahepatic metastasis (no vs yes)	1.089	0.568–2.088	0.789			
Tumor size (<10 cm vs ≥10 cm)	1.112	0.605–2.042	0.733			
AFP (≥400 ng/mL vs <400 ng/mL)	1.149	0.626–2.106	0.654			
Treatment lines(1 vs 2)	2.18	1.105–4.284	0.025	3.215	1.520–6.951	0.002

HR, hazard ratio; CI, confidence interval; ECOG PS, eastern cooperative oncology group performance status; AFP, alpha fetoprotein; BCLC, barcelona clinic liver cancer; HBV, hepatitis B virus; PVTT, portal vein tumor thrombus.

## Discussion

In this study, the ORR and DCR of patients receiving HAIC combination PD-1 antibody and lenvatinib were 36.1 (RECIST 1.1)/57.4% (mRECIST) and 82.0%, respectively. In the first-line treatment, the ORR and DCR were 44.7/66.0% and 83.0%. In the second-line group, the ORR and DCR were 7.1/28.6% and 78.6%. The median PFS of the whole population was 6.0 months, the median PFS of first-line treatment was 6.7 months, and the median PFS of second-line treatment was 4.3 months. It is worth mentioning that based on the mRECIST criteria, 19.1% of patients achieved CR in the first-line treatment. In addition, AFP level decreased significantly after the first triple therapy, which can effectively control intrahepatic lesions and has less effect on liver function. Although the triple therapy increases the incidence of toxic reactions, it is controllable. Kaplan–Meier survival analysis and COX multivariate analysis showed that the factors affecting PFS were the number of treatment lines and age.

Based on RECIST 1.1 criteria, the first-line treatment ORR was 44.7% and the DCR was 83.0%, which was similar to the ORR (18.3–58.3%) and DCR (18.3–90.0%) of patients with advanced HCC who had previously received lenvatinib, pembrolizumab plus lenvatinib, and HAIC combined with lenvatinib (11–13). Based on mRECIST criteria, the ORR and DCR of first-line treatment patients were 66.0 and 83.0%, similar to the previous research results of HAIC combined with PD-1 antibody and molecular targeted agent (ORR 40 to 100%, DCR 77.6–100%) (9, 10, 14–16). In this study, however, the PFS observed in the first-line treatment group (6.7 months) was significantly worse than that in the previous study (8.8–11.1 months) (10, 15, 16). This may be related to the following reasons: (1) The follow-up time is short; up to now, 29.8% of patients have not progressed; (2) the proportion of patients with cancer thrombus in the main portal vein was 68.1% (15.5–35.6% in the previous study); (3) chronic hepatic B virus infection was the predominant cause of HCC (previously studied as 53.6–82.2%). For HBV-positive hepatocellular carcinoma, however, studies have reported that HAIC may be inferior than HCV (17, 18). (4) The proportion of extrahepatic metastasis was 68.1% (22.5–33% in previous studies). In terms of second-line treatment, ORR was 7.1% (RECIST 1.1), DCR was 78.6%, and PFS was 4.7 months. Because the current data of HAIC triple therapy as second-line treatment for HCC are limited, it is difficult to conduct in-depth comparison. Compared with patients with advanced HCC receiving regorafenib, PD-1 antibody, and HAIC as second-line treatment, there is no significant benefit in ORR (11–20%, recist1.1), DCR (58–66%), and PFS (3.0–4.9 months) (8, 19–21). The utility of triple therapy in second-line treatment needs to be further explored in the future.

In terms of safety, adverse reactions were controllable. The overall incidence of adverse events related to HAIC combined with PD-1 inhibitor and lenvatinib was similar to previous studies (10, 15, 16). The overall incidence of TRAE was 75.4% (46/61). Common adverse events included neutropenia 50.8% (31/61), abdominal pain 45.9% (28/61), ALT increase 39.3% (24/61), and thrombocytopenia 36.1% (22/61). A total of 16 patients (26.2%) experienced serious adverse events (grades 3–4). The common serious adverse events included abdominal pain 8.2% (5/61), neutropenia 6.6% (4/61), thrombocytopenia 4.9% (3/61), ALT increase 3.3% (2/61), and AST increase 3.3% (2/61). The common adverse event associated with HAIC is myelosuppression after chemotherapy, which is characterized by the decline of neutrophils and platelets. According to the review of 4,580 patients with HAIC complications, 5-fluorouracil was linked to myelosuppression toxicity (22). Another common adverse reaction is abdominal pain. Previous research has revealed that the infusion of oxaliplatin is the major cause of HAIC discomfort, which may be connected to the oxaliplatin infusion time, the diameter of the hepatic artery, and the oxaliplatin manufacturer (23). Prolonging the infusion time and arterial infusion of lidocaine are the effective means to reduce pain. Hypothyroidism (18.0%), mostly grades 1–2, was the most common immune-related TRAE, and there were no serious adverse events. Since the manifestations of hypothyroidism are fatigue, chills, constipation, and lower limb edema, which are difficult to distinguish from the symptoms of the tumor itself, it should be closely observed and identified as soon as possible in clinical practice. In addition, two patients developed immune-related hepatitis. Their liver function improved after 2 weeks of glucocorticoid therapy and the discontinuation of PD-1 antibody treatment. According to relevant clinical experience and literature reports, for patients with immunological hepatitis, steroid treatment should be completely stopped before starting immunotherapy again; otherwise, the risk of rebound will be increased (24, 25). The most common TRAE with lenvatinib was hypertension (36.1%), similar to the results of previous studies. Therefore, HAIC combined with PD-1 antibody and lenvatinib in the treatment of advanced HCC did not significantly increase serious adverse reactions, mainly manifested in chemotherapy-related adverse reactions and abdominal pain, which was relatively safe.

The synergistic antitumor mechanisms might explain the remarkable tumor response rates seen in triple therapy patients. Lenvatinib increases T-cell infiltration in tumors and inhibits immunosuppressive cells in the tumor microenvironment by inhibiting VEGFR, as well as lowering monocytes and macrophages, increasing the proportion of gamma interferon-induced CD8 + T cells, and boosting immunotherapy efficacy (26). According to the KEYNOTE-524 trial, pembrolizumab plus lenvatinib showed excellent antitumor activity in patients with HCC (ORR was 46%) (27). Lenvatinib is an anti-angiogenic agent that helps chemotherapy drugs reach the lesion by normalizing tumor blood vessels (28). The LEOPARD study showed a promising ORR (64.7%) (13). It has been proven that 5-FU and oxaliplatin can induce ICIs and reverse the resistance to the immunotherapy. Adding PD-1 antibody can suppress the secretion of TGF-β, increase inflammatory cytokines, and promote the efficacy against PD-1 (29–31). As demonstrated previously, both the overall response rate (83.0 vs. 66%; *p* = 0.006) and intrahepatic response rate (85.0 vs. 74%; *p* = 0.045) in HAIC coupled with PD-1 antibody group were higher than the HAIC alone group (31).

There were several limitations in this study. First, this is a single-center retrospective research that is prone to selection bias and can be impacted by specific treatment approaches. Second, since to the short follow-up time, only 45 patients (73.8%) reached the PFS endpoint. Particularly, in first-line therapy, approximately 30% of patients did not progress and require substantial follow-up. Third, the majority of the patients in this study were infected with the hepatitis B virus, and the effectiveness of triple treatment for patients with HCC of other etiologies has to be further examined. Finally, we did not undertake a subgroup analysis of the PD-1 antibody employed in patients. Therefore, we will continue to explore the association between different categories of immune checkpoint inhibitors and survival outcomes.

## Conclusion

The results of this study indicated that HIAC combined with lenvatinib and PD-1 antibody may have a potential benefit and well-tolerated toxicity in patients with advanced HCC.

## Data availability statement

The original contributions presented in this study are included in the article/supplementary material, further inquiries can be directed to the corresponding author.

## Ethics statement

Written informed consent was obtained from the individual(s) for the publication of any potentially identifiable images or data included in this article.

## Author contributions

YX and SF conceived the study and wrote the manuscript. YX conducted the work. YX, DL, and SH obtaining and analyzed the data. YM and JW reviewed the manuscript. All authors listed have read and approved the manuscript.
